# The Mechanism of Porcelain Toughened by Activated Kaolinite in a Lower Sintering Temperature

**DOI:** 10.3390/ma15113867

**Published:** 2022-05-28

**Authors:** Shaomin Lin, Yaling Yu, Yue Tan, Huan Yang, Mingfeng Zhong, Chenyang Zhang, Zhijie Zhang, Yunying Wu

**Affiliations:** 1 School of Materials Science and Engineering, Hanshan Normal University, Chaozhou 521041, China; lsm678@hstc.edu.cn (S.L.); yaxiulingwu@163.com (Y.Y.); yanghuan@hstc.edu.cn (H.Y.); 2 Chaozhou Branch of Chemistry and Chemical Engineering Guangdong Laboratory, Chaozhou 521041, China; 3 School of Materials Science and Engineering, South China University of Technology, Guangzhou 510641, China; grandnavy@sina.com (Y.T.); mfzhong@scut.edu.cn (M.Z.); 4 Guangdong Chaoshan Institute of Higher Education and Technology, Chaozhou 521041, China; yunyingwu@hstc.edu.cn

**Keywords:** porcelain, activated kaolinite, citric acid, sintering temperature

## Abstract

A high sintering temperature is usually required to acquire excellent performance in the ceramic industry, but it results in high fuel consumption and high pollution. To reduce the sintering temperature and to toughen the porcelain, a self-produced sintering additive of citric acid activated kaolinite was added to the raw material; X-ray powder diffraction (XRD), transmission electron microscope (TEM), scanning electron microscope (SEM), and thermal gravity analysis and differential scanning calorimetry (TG-DSC) were used to characterize the samples, and the toughening mechanism was discussed. The citric acid activated kaolinite obtained high activity and a large specific surface area. After introducing the activated kaolinite, the bending strength of porcelain at 1270 °C increased from 100.08 MPa to 124.04 Mpa, which was 11.45% higher than that of porcelain without activated kaolinite at 1350 °C. The results of XRD revealed that the content of mullite increased and the quartz decreased at 1270 °C, and the well-distributed needle-like mullite was observed in the images of SEM with the addition of citric acid activated kaolinite. The TG-DSC results indicated adding activated kaolinite to porcelain raw materials reduced the formation of mullite to 994.6 °C. The formation of mullite in a lower temperature served as mullite seeds in a green body during firing, and it enhanced the growth of mullite. These contributed to the appropriate phase composition and the excellent microstructure of porcelain. Thus, the distinguished mechanical performance of porcelain was obtained. Moreover, the sintering additive had no adverse effect on the porcelain body as citric acid-activated kaolinite was one of the main components of the porcelain raw material.

## 1. Introduction

The most significant development in the history of ceramics was the production of a vitrified, translucent porcelain body in ancient China [1]. Today, porcelain is studied widely by different researchers in different countries due to its economy, practicality, and excellent performance [2,3,4,5,6]. The raw materials used for the body compositions of porcelain can be divided into three groups of minerals: clay, feldspar, and quartz. Each has its own function: clay mineral gives plasticity to the body, while the complementary non-plastic includes both melting and structural minerals. Kaolinite, feldspar, and quartz of the triaxial porcelain compositions were widely used and studied. The kaolinite plays a significant role in the mechanical properties of porcelain, as the mullite formation from kaolinite during firing remarkably affects the strength of porcelain. A high sintering temperature is usually required to acquire an appropriate amount of the mullite phase and the liquid phase [7,8,9]. Therefore, the porcelain industry is characterized by high fuel consumption and pollution.

One way of reducing the sintering temperature of porcelain is to add a sintering additive to the raw materials. Several sources of research have reported that the sintering temperature decreased by mixing metal oxides or other fluxing agents into porcelain, whereas these expensive sintering aids are hardly applied to porcelain production [10,11,12,13,14]. O. Turkmen reduced the firing temperature of 25 °C of hard porcelain by 1 wt% wollastonite addition, but the bending strength decreased and the porosity increased [15]. S. Akpinar reported that the firing temperature decreased by approximately 50 °C by adding 1 wt% of colemanite treated by microwave-assisted calcination [16], which also consumed additional energy. An economical and an environmentally friendly sintering additive is urgently required in ceramics production.

In this paper, the acid activated kaolinite was prepared and used as a sintering additive. In the previous work, we effectively activated kaolinite by oxalic and citric acid [17], a low molecular weight organic acid ubiquitous in soils and sediments, and we produced mullite from the citric activated kaolinite at a lower temperature [18]. In the present work, the kaolinite activated by citric acid was used as a sintering additive. The morphology and the mechanical performance of the green body were investigated. The results may provide some implication in lowering the sintering temperature of ceramics and achieving energy savings.

## 2. Materials and Methods

### 2.1. Preparation

**Figure 1:**
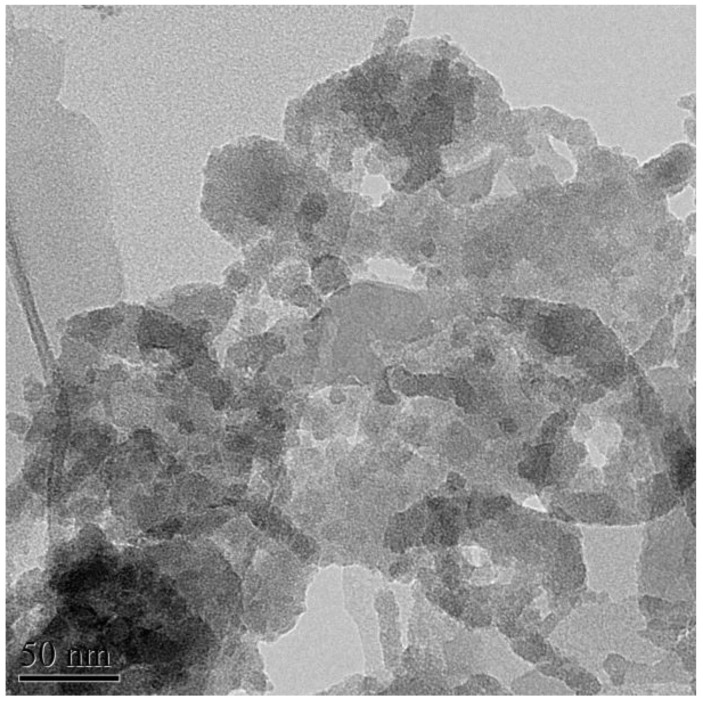
The morphology of the kaolinite activated by citric acid.

Kaolinite (Guangdong Changlong Ceramic Co., Ltd., Meizhou, China) activated by citric acid (C_6_H_8_O_7_·H_2_O, 99%, Fuchen Chemical Reagents Factory, Tianji. China), and the preparation of the activated kaolinite was presented in the previous work of the authors in 2017 [18]. The porcelain raw materials were marked as P, and the raw material (Guangdong Changlong Ceramic Co., Ltd., Meizhou, China) mixed with 5 wt% activated kaolinite was marked as PK. The chemical compositions of P and PK are shown in Table 1. The morphology of activated kaolinite is shown in Figure 1.

**Figure 2:**
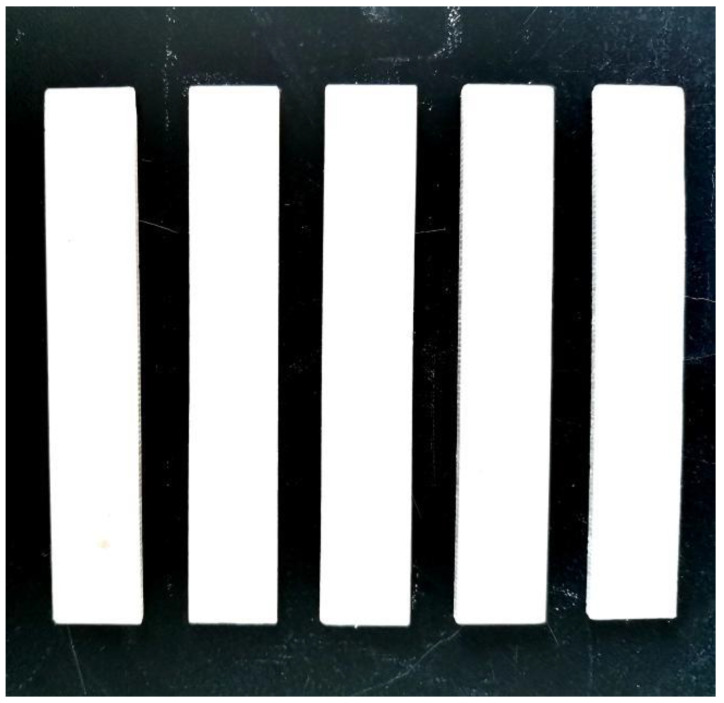
The image of the laboratory specimens obtained.

The powders of P and PK were ball milled (QM-3SP2 planetary ball mill, Nanjing Nanda Instrument Plant Co., Ltd., Nanjing, China) in distilled water for 6 h using polyethylene bottles with zirconia ball media. After drying at 85 ± 1 °C in air, the powders were ground in an agate mortar and passed through a 200 mesh nylon sieve. Specimens were fabricated by dry-pressing moulding (120 mm (L) × 20 mm (W) × 3 mm (H)) at 20 MPa (Figure 2). The green specimens were fired in air at 1270 °C, 1350 °C in an electric furnace at a heating rate of 5 °C/min in air, and then they remained at these temperatures for 2 h before cooling the furnace. The porcelain from the raw material was marked M1, and the porcelain from the materials mixed with 5% activated kaolin was marked M2.

### 2.2. Methods

The chemical compositions of the samples were analyzed by XRF Spectrometry (PANalytical Axios PW4400, Amsterdam, The Netherlands) with a fusion dissolution technique.

The thermal behavior of the samples was investigated by thermal gravity analysis and differential scanning calorimetry (TG-DSC) in air, by a NETZSCH STA 449C (NETZSCH-Gerätebau GmbH, Selb, Germany) thermal analyzer with a heating rate of 10 °C/min between 25 °C and 1100 °C.

The crystalline phase of samples was identified by the X-ray powder diffraction technique, using a Rigaku D/Max-IIIA X-ray diffractometer (Rigaku Corporation, Tokyo, Japan) operated at 30 kV and 10 mA with Cu Kα radiation and a curved graphite secondary monochromator covering 2θ between 10 and 70° with a step width of 0.02° and 0.1 s data collection per step.

The morphology of the raw material was examined by using a transmission electron microscope (JEM-2100F, Japan Electronics Co., Ltd., Tokyo, Japan) operated at 200 kV. The micrographs of the sample were obtained from powdered samples deposited on a holey Cu grid.

The morphology of the samples was examined using a Quanta 200 scanning electron microscope (FEI, Hillsboro, America) with a field emission gun operating normally at 15–20 kV of acceleration voltage in a high vacuum environment. The sintered samples were etched by 20% (w/w) hydrofluoric acid solution for 2 min before a Platinum sputtering treatment.

The apparent porosity (%) and the bulk density (g/cm^3^) of the samples was measured based on Archimedes’ principle according to Equation (1) and Equation (2), respectively. The water absorption (%) of the samples was obtained by the GB/T 3299-2011 according to Equation (3). Five samples from each group were measured to reliably obtain apparent porosity, water absorption, and bulk density [19]. The weight (g) of the samples was performed using a precision electronic balance (FA3204B, Tianmei, Shanghai, China) with a density determination device (MD, Tianmei, Shanghai, China).

P_a_ = (1 − (m_1_ − m_2_)/(m_3_ − m_2_)) × 100% (1)

D_b_ = *ρ* × m_1_/(m_3_ − m_2_) (2)

W_a_ = (m_3_ − m_1_)/m_1_ × 100% (3)

m_1_ = Dry weight (g) of samples placed in the drying oven for 24 h.

m_2_ = Float weight (g) of samples in water.

m_3_ = Wet weight (g) of samples after complete immersion in distilled water under constant vacuum for 60 min.

*ρ* = Density of distilled water (1 g/cm^3^).

P_a_ = Apparent porosity (%).

W_a_ = Water absorption (%).

D_b_ = Bulk density (g/cm^3^).

The three-point bending test was performed using a microcomputer control universal material test machine (TFW-100B, Jinan Xinguang Test Machine Manufacturing Co., Ltd., Jinan, China). The three-point bending span was 50 mm, and the loading rate was 0.5 mm/min. The recorded values of the fracture load were used to calculate the bending strength (σf):

σf = (3 × F_max_ × L)/(2 × b × d^2^) (4)

where F, L, b, and d are the maximum load (N), support span (mm), the width of the tested beam (mm), and the thickness of the tested beam (mm), respectively. Five samples were measured to obtain reliable bending strength.

## 3. Results and Discussion

### 3.1. The Properties of the Sintered Samples

Table 2 shows the apparent porosity, water absorption, bulk density, and bending strength of the samples. By increasing the sintering temperature, the bulk density and bending strength increased, and the apparent porosity decreased for M1 and M2. This was probably due to the formation of a more liquid phase and ejection of more bubble, and the formation and growth of mullite crystal at a higher temperature. It was noted that the bulk density and the bending strength of M2 at 1270 °C was higher than that of M1 at 1350 °C, which was the optimum sintering temperature of raw material. This indicated that the addition of activated kaolinite facilitated to increase the compactness and the mechanical properties of porcelain at a lower temperature.

### 3.2. XRD Analysis

**Figure 3:**
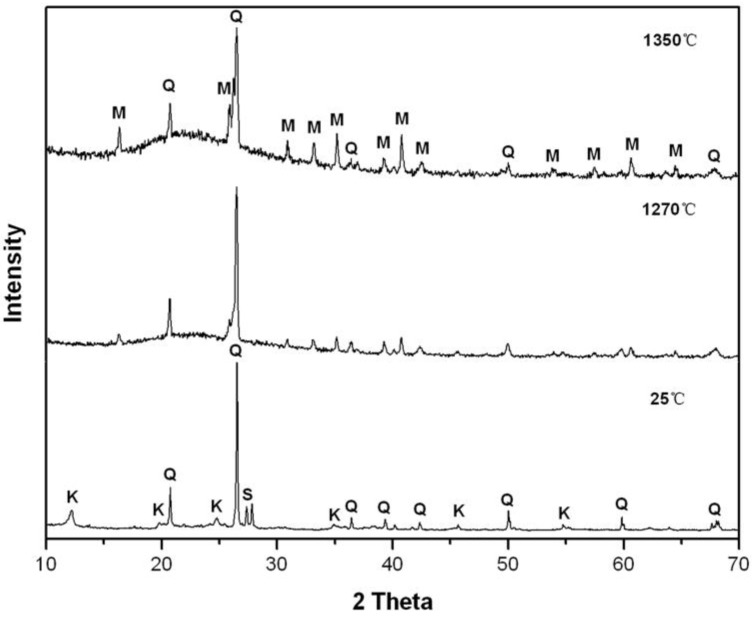
XRD patterns of M1 at 1270 °C and 1350 °C for 2 h; Q: quartz, M: mullite, K: kaolinite, S: sanidine.

**Figure 4:**
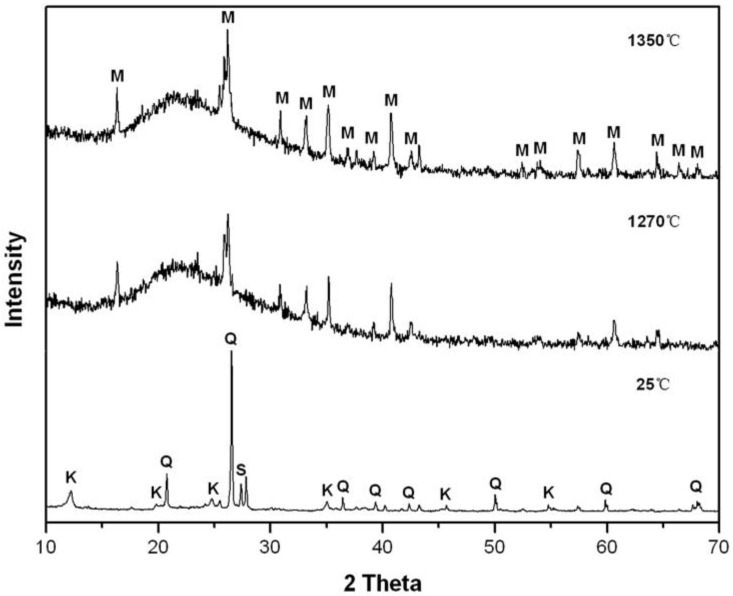
XRD patterns of M2 at 1270 °C and 1350 °C for 2 h; Q: quartz, M: mullite, K: kaolinite, S: sanidine.

Figure 3 and Figure 4 exhibit the XRD patterns of the porcelain raw body and the porcelain body mixed with activated kaolinite at 25 °C and being sintered at 1270 °C and 1350 °C for 2 h. At 25 °C, the porcelain raw body contained mainly quart, kaolinite, and sanidine, and the XRD pattern has no obvious change after adding the 2.5% additive; at 1270 °C and 1350 °C, both kaolinite and sanidine disappeared in M1 and M2, due to the kaolinite decomposition and the sanidine fusion. The XRD reflection intensity for quartz decreased, while the mullite phase increased in both M1 and M2, with increasing sintering temperature, probably due to the formation of much more mullite and the fusion of quartz at a higher temperature. The mullite phase plays an important role in the structure and the function of porcelain due to its low thermal expansion, good chemical and thermal stability, and superior creep resistance [20,21,22,23,24]. An appropriate amount of the mullite phase was beneficial to improve the mechanical performance of the green body. Therefore, the bending strength of porcelain increased with the increase of temperature (Table 2).

With the addition of a sintering additive, the background peaks at 1270 °C and 1350 °C, attributed to the amorphous phase [25,26,27], obviously increased; the XRD reflection intensity of the quartz phase was higher than that of the mullite phase in M1 at 1270 °C and 1350 °C (Figure 3). The XRD reflection intensity of the mullite phases increased, while the quartz phase almost disappeared in M2 (Figure 4) at 1270 °C and 1350 °C. This indicated that the addition of activated kaolinite improved the formation of the mullite phase and the glass phase. The more amorphous phase and the mullite crystal may be responsible for the higher bulk density and bending strength of M2 at 1270 °C and 1350 °C.

### 3.3. SEM Analysis

**Figure 5:**
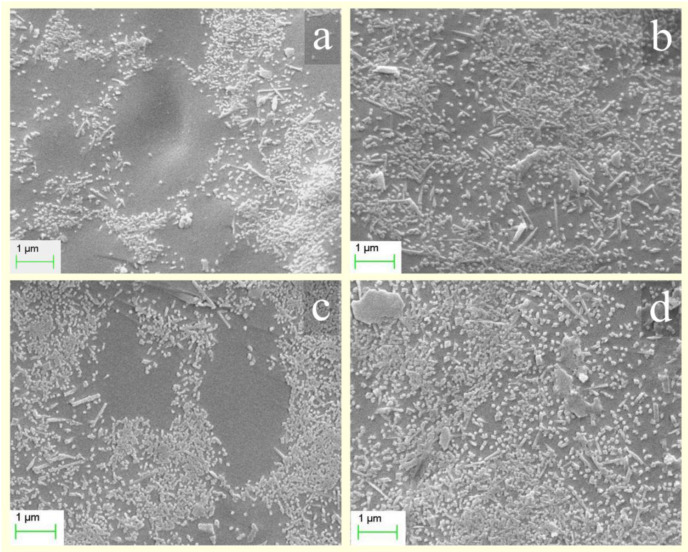
SEM pictures of M1 at (**a**) 1270 °C and (**c**) 1350 °C; SEM pictures of M2 at (**b**) 1270 °C and (**d**) 1350 °C.

The SEM pictures of the fractured surface of M1 and M2 are shown in Figure 5. Numerous tiny needle-shaped crystallites were formed in the porcelain body, which were identified as mullite by XRD (Figure 3 and Figure 4). At 1270 °C, a small amount of mullite crystal was found in M1 and the distribution of mullite was non-uniform (Figure 5a). By increasing the temperature to 1350 °C, the content of mullite increased but the distribution of mullite was still uneven, and the length of the mullite crystal was mainly less than 1 μm (Figure 5c). After mixing with activated kaolinite, the amount and the length of the mullite increased in M2 at 1270 °C, and the distribution of the mullite phase was relatively uniform (Figure 5b). The higher bending strength of M2 (Table 2) was attributed to these interlocked mullite crystals [28]. This suggested that mixing citric acid activated kaolinite as a sintering additive into porcelain raw material could achieve the desired microstructure and size of mullite crystal at a lower sintering temperature. In addition, the size of the crystals and the main crystal phase were stably maintained as the sintering temperature increased from 1270 °C to 1350 °C; the composition of the the crystal phase changed slightly (Figure 4) and the mullite grain content even increased (Figure 5b,d). This indicated the activated kaolinite mixed in the porcelain material had a widened maturing range, which was suitable to the production of industry. 

### 3.4. TG-DSC Analysis

**Figure 6:**
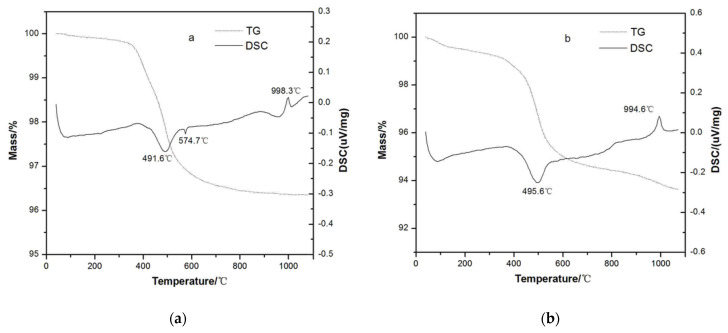
TG−DSC curves of (**a**) M1 and (**b**) M2.

Figure 6 shows the TG and the DSC curves of raw materials with and without activated kaolinite. The mass losses of M1 were 0.16%, 3.25%, and 0.77% (Table 3, Figure 6a), corresponding to the release of the free and the absorbed water, loss of interlayer water and organic matters, and volatilization of the solid solution at a high temperature, respectively; while for M2, the mass loss values of 0.76%, 4.68%, and 1.01% (Figure 6b) were larger than that of M2, possibly due to the presence of some organic acid and metal-organic complex. After mixing the activated kaolinite, the endothermic peak attributed to the lattice rearrangement decreased from 998.3 °C to 994.6 °C. This suggested that mixing activated kaolinite to porcelain raw materials facilitated the formation of mullite at a lower temperature, which may be attributed to the high activity of kaolinite after treatment. The in situ formation of mullite from activated kaolinite at a lower temperature may play a role in crystal seeds, which facilitated the formation of much more mullite during the firing process.

## 5. Conclusions

A total of 2.5 wt% self-produced sintering additive of citric acid-activated kaolinite was mixed into porcelain raw material. After mixing activated kaolinite, the bending strength of the green body at 1270 °C increased to 124.04 MPa, which was even 11.45% higher than that of raw material at optimum temperature of 1350 °C. The XRD analyses showed the XRD reflection intensity of mullite and the glass phase increased and the quartz decreased after mixing with activated kaolinite at 1270 °C. In addition, SEM pictures of the porcelain body showed that the length of mullite crystals increased and the distribution of mullite was even after mixing activated kaolinite. The TG-DSC results indicated that adding activated kaolinite to porcelain raw materials facilitated the formation of mullite at a lower temperature. This indicated that the addition of activated kaolinite optimized the phase composition and the microstructure of the porcelain body. In addition, the activated kaolinite had no adverse effect, such as a mismatch of the firing system between the porcelain body and the sintering additive because it is one of the compositions of the porcelain body.

The improvement of performance for activated kaolinite mixed porcelain raw material at a lower temperature is probably explained by the following two reasons. On the one hand, the addition of highly active kaolinite promoted the formation of a more liquid phase in the green body, and moderate liquid benefitted the decrease in the apparent porosity, and it increased the bulk density, resulting in an increase in compactness. On the other hand, the in situ formation of mullite from activated kaolinite at a lower temperature may play a role in crystal seeds, which facilitated the formation of much more mullite during the firing process. The results are of interest in producing ceramics at reduced sintering temperatures and consequential energy saving for the sintering process.

## Figures and Tables

**Table 1:** Chemical composition (wt%) of porcelain raw kaolinite (P) and raw materials mixed with 5 wt% activated kaolinite (PK).

Samples	SiO_2_	Al_2_O_3_	Fe_2_O_3_	CaO	MgO	K_2_O	Na_2_O	Ti_2_O	Loss of Ignition
P	70.48	21.39	0.97	1.48	0.19	0.20	0.41	0.31	4.58
PK	69.58	22.09	0.94	1.41	0.19	0.23	0.40	0.30	4.87

**Table 2:** Properties of porcelain raw materials with and without activated kaolinite sintered at different temperatures.

Temperature/°C	Samples	Apparent Porosity/%	Water Absorption/%	Bulk Density/(g·cm^−3^)	Bending Strength/MPa
1270	M_1_	2.93 ± 0.042	1.68 ± 0.021	1.75 ± 0.037	100.08 ± 3.181
1270	M_2_	0.15 ± 0.003	0.11 ± 0.003	1.82 ± 0.025	124.04 ± 3.225
1350	M_1_	0.14 ± 0.002	0.09 ± 0.001	1.77 ± 0.031	111.30 ± 2.667
1350	M_2_	0.09 ± 0.001	0.05 ± 0.001	1.90 ± 0.019	137.66 ± 2.207

M_1_: the raw material; M_2_: the material mixed with 5% activated kaolin.

**Table 3:** The summary of TG and DSC data of P and PK.

Samples	Mass Loss (%) at Temperature Range (°C)			The Temperature (°C) of the Main Peaks	
	RT–300	300–700	700–1100	Dehydroxylation	Formation of Mullite
P	0.16	3.25	0.23	491.6	998.3
PK	0.76	4.68	1.01	495.6	994.6

## Data Availability

Not applicable.
